# 03. Immunogenicity and Safety of a Quadrivalent Meningococcal Conjugate Vaccine (MenACYW-TT) Administered as a Booster Dose in Adults and Adolescents Vaccinated Against Meningococcal Disease 3 - 6 Years Earlier

**DOI:** 10.1093/ofid/ofab466.206

**Published:** 2021-12-04

**Authors:** James Peterson, Carmen Deseda, Katie Julien, Betzana Zambrano, Germán Áñez, Sue Jiayuan, Judy Pan, Habiba Arroum, Kucku Varghese, Emilia Jordanov, Mandeep S Dhingra

**Affiliations:** 1 J Lewis Research, Salt Lake City, Utah; 2 Caribbean Travel Medicine Clinic, San Juan, Puerto Rico; 3 Sanofi Pasteur, Montevideo, Montevideo, Uruguay

## Abstract

**Background:**

Booster doses of meningococcal conjugate vaccines may induce long-term protection against invasive meningococcal disease. MenACYW-TT [MenQuadfi^®^] is a quadrivalent meningococcal conjugate vaccine, licensed for use in ages 2 years and older in USA. The vaccine is also licensed in ages 12 months and older in EU and other countries.

**Methods:**

A phase IIIb study (NCT04084769) was conducted to evaluate the persistence of immune response in adults and adolescents primed 3-6 years earlier with either MenACYW-TT or MCV4-CRM (Menveo^®^) and, safety and immunogenicity of MenACYW-TT when administered as a booster dose with or without concomitant administration with MenB vaccines (Bexsero^®^ and Trumenba^®^). Serum bactericidal assays with human complement (hSBA) and baby rabbit complement (rSBA) were used to measure antibodies against vaccine serogroups at baseline (Day 0 [D0]), D06 (in a subset) and 30 days post-vaccination (D30). Safety data were collected up to 6 months post-vaccination.

**Results:**

At D0, the GMTs were higher in subjects primed with MenACYW-TT vs MCV4-CRM for serogroups C, Y and W, and were comparable for serogroup A. At D0, all hSBA GMTs were higher than those observed pre-priming dose, suggesting persistence of immunity. Sufficiency of hSBA seroresponse ( >75%) was demonstrated following administration of MenACYW-TT booster dose regardless of the priming vaccine administered 3-6 years earlier. Vaccine seroresponse in a subset of participants at D06 ranged from 77.8% (95%CI 62.9%; 88.8%) for serogroup A to 97.8% (88.5%; 99.9%) for serogroup W suggesting a quick onset of immune response post-booster. Post-vaccination (D30) hSBA GMTs were comparable for serogroups A, Y and W regardless of the nature of the priming vaccine and were higher for serogroup C in subjects primed with MenACYW-TT vaccine. The MenACYW-TT booster dose was well-tolerated and had similar safety profiles regardless of the priming vaccine. The safety profiles were comparable regardless of the MenB vaccine co-administered with MenACYW-TT vaccine.

**Conclusion:**

MenACYW-TT used as priming vaccine was able to demonstrate persistence of immune response 3-6 years later. MenACYW-TT elicits robust booster responses in adults and adolescents primed with MenACYW-TT or MCV4-CRM

**Disclosures:**

**Betzana Zambrano, MD**, **Sanofi Pasteur** (Employee) **Germán Áñez, MD**, **Sanofi Pasteur** (Other Financial or Material Support, Former employee) **Sue Jiayuan, MSc**, **Sanofi Pasteur** (Independent Contractor) **Judy Pan, PhD**, **Sanofi Pasteur** (Employee) **Habiba Arroum, MD**, **Sanofi Pasteur** (Employee) **Kucku Varghese, PhD**, **Sanofi Pasteur** (Employee) **Emilia Jordanov, MD**, **Sanofi Pasteur** (Employee, Shareholder) **Mandeep S. Dhingra, MD**, **Sanofi Pasteur** (Employee, Shareholder)

